# Two members of *TaRLK* family confer powdery mildew resistance in common wheat

**DOI:** 10.1186/s12870-016-0713-8

**Published:** 2016-01-25

**Authors:** Tingting Chen, Jin Xiao, Jun Xu, Wentao Wan, Bi Qin, Aizhong Cao, Wei Chen, Liping Xing, Chen Du, Xiquan Gao, Shouzhong Zhang, Ruiqi Zhang, Wenbiao Shen, Haiyan Wang, Xiue Wang

**Affiliations:** The State Key Laboratory of Crop Genetics and Germplasm Enhancement, Cytogenetics Institute, Nanjing Agricultural University/JCIC-MCP, Nanjing, Jiangsu 210095 China; State Key Laboratory of Cotton Biology, Institute of Cotton Research of Chinese Academy of Agricultural Sciences, Anyang, Henan 455000 China; Key Laboratory of Biology and Genetic Resources of Rubber Tree, Ministry of Agriculture, Rubber Research Institute, Chinese Academy of Tropical Agricultural Sciences, Danzhou, Hainan 571737 China; College of Life Sciences, Nanjing Agricultural University, Nanjing, Jiangsu 210095 China

**Keywords:** *Triticum aestivum* L, Powdery mildew, Receptor-like kinase, Transgenic wheat

## Abstract

**Background:**

Powdery mildew, caused by *Blumeria graminearum* f.sp. *tritici* (*Bgt*), is one of the most severe fungal diseases of wheat. The exploration and utilization of new gene resources is the most effective approach for the powdery mildew control.

**Results:**

We report the cloning and functional analysis of two wheat *LRR-RLKs* from *T. aestivum* c.v. Prins- *T. timopheevii* introgression line IGV1-465, named *TaRLK1* and *TaRLK2*, which play positive roles in regulating powdery mildew resistance in wheat. The two *LRR-RLKs* contain an ORF of 3,045 nucleotides, encoding a peptide of 1014 amino acids, with seven amino acids difference. Their predicted proteins possess a signal peptide, several LRRs, a trans-membrane domain, and a Ser/Thr protein kinase domain. In response to *Bgt* infection, the *TaRLK1*/*2* expression is up-regulated in a developmental-stage-dependent manner. Single-cell transient over-expression and gene-silencing assays indicate that both genes positively regulate the resistance to mixed *Bgt* inoculums. Transgenic lines over-expressing *TaRLK1* or *TaRLK2* in a moderate powdery mildew susceptible wheat variety Yangmai 158 led to significantly enhanced powdery mildew resistance. Exogenous applied salicylic acid (SA) or hydrogen peroxide (H_2_O_2_) induced the expression of both genes, and H_2_O_2_ had a higher accumulation at the *Bgt* penetration sites in RLK over-expression transgenic plants, suggesting a possible involvement of SA and altered ROS homeostasis in the defense response to *Bgt* infection. The two *LRR-RLK*s are located in the long arm of wheat chromosome 2B, in which the powdery mildew resistance gene *Pm6* is located, but in different regions.

**Conclusions:**

Two members of *TaRLK* family were cloned from IGV1-465. *TaRLK1* and *TaRLK2* contribute to powdery mildew resistance of wheat, providing new resistance gene resources for wheat breeding.

**Electronic supplementary material:**

The online version of this article (doi:10.1186/s12870-016-0713-8) contains supplementary material, which is available to authorized users.

## Background

Upon the detection of pathogen, plants activate innate immune system to defend pathogen attack. Receptor-like kinase (RLK) membrane proteins serve as pattern recognition receptors (PRRs) and play essential roles in detecting pathogen-associated molecular patterns (PAMPs). They initiates basal and broad-spectrum defense, known as pattern triggered immunity (PTI). RLKs have been identified in many plant species and have been implicated in regulating the processes of plant growth, development, and responses to biotic and/or abiotic stresses. Most of the RLKs identified as being involved in plant defense are of the LRR-RLK class including the rice Xa21 protein and the *Arabidopsis* Flagellin Sensitive 2 (FLS2) and bacterial translation elongation factor EF-Tu receptor (EFR). Recent identification in rice of a lysine-motif (LysM) receptor kinase involved in the recognition of the fungal elicitor chitin [1] and a lectin receptor kinase (LecRK) involved disease resistance indicates that other RLK classes may also play important or overlapping roles in plant defense and pathogen recognition [2]. FLS2 and EFR act as PRRs to detect PAMPs and trigger immune responses in *A. thaliana*. The chitin elicitor receptor kinase 1 of *Arabidopsis* (AtCERK1) directly binds chitin through its lysine motif (LysM)-containing ectodomain to activate immune responses [3]. The rice gene *Xa21,* which codes for an LRR-RLK with 23 extracellular LRR repeats of 24 amino acids each and an intracellular serine/threonine kinase domain, confers race-specific resistance to *Xoo* (*Xanthomonas oryzae* pv *oryzae*) [4]. *Xa21* is developmentally controlled: juvenile rice plants challenged with *Xoo* are less resistant than older plants [5]. *Xa3/Xa26* also encodes an LRR-RLK, but does not appear to be developmentally regulated, as both juvenile and adult plants exhibit resistance against *Xoo* [6]. The tomato resistance gene *Cf*, which encodes a protein containing extracellular LRR domains but lack the cytoplasmic protein kinase domain, also confer a race-specific resistance to *Cladosprium fulvum* [7]. Up to now, only a few RLKs have been functionally identified, which is even more so in common wheat (*Triticum aestivum* L.).

Wheat powdery mildew, caused by a biotrophic fungus *Blumeria graminis* f.sp. *tritici* (*Bgt*) is one of the most serious diseases of common wheat. Breeding and utilizing wheat varieties with powdery mildew resistance are widely-accepted strategies for the disease control. To date, among the 45 identified powdery mildew resistance genes [8], only *Pm3* together with its multiple alleles, *Pm38*, *Pm8* and a key member of *Pm21* have been cloned [9, 10]. *Pm3* is located on the short arm of wheat chromosome 1A [11] and 15 functional alleles have been identified at this locus (*Pm3a* to *Pm3g*, *Pm3k* to *Pm3r*). Further results reveal that the *Pm3* alleles confer race-specific resistance to different subsets of *Bgt* races [9, 12, 13]. Recent research show that the rye *Pm8* is an ortholog of *Pm3*, which suppresses the *Pm8*-mediated powdery mildew resistance in lines containing *Pm8* [14]. The *Pm21* gene is located on the chromosome 6VS of *Haynaldia villosa*, which is a diploid wheat relative. Cao et al. reported that a key member of *Pm21* encoding a putative serine and threonine protein kinase conferred broad-spectrum resistance to powdery mildew in wheat [9, 12, 13].

A series of *Triticum aestivum* c.v. Prins- *Triticum timopheevii* (2n = 4x = 28, genome AAGG) introgression lines with different introgressed 2G chromosome fragment sizes have been developed and characterized [15–18]. The powdery mildew resistance gene *Pm6*, whose effects depend on developmental stages, is located on the long arm of chromosome 2G. The developed introgression lines all show powdery mildew resistance, especially at their adult stages, and have been widely used in wheat breeding programs. However, the genetic mechanism for powdery mildew resistance of these introgression lines is still not clear. In the present study, two members from the *LRR-RLK* cluster were cloned from *T. aestivum* c.v. Prins- *T. timopheevii* introgression line IGV1-465. The two genes both exhibit a developmentally dependent expression manner in response to *Bgt* infection. Their transient and stable transformation improved the powdery mildew resistance of the susceptible wheat variety Yangmai 158, while knockdown of the genes by transient gene silencing compromised the resistance level of the resistant lines, suggesting that both genes are involved in powdery mildew resistance in wheat.

## Methods

### Plant materials

The powdery mildew susceptible Swedish common wheat variety Prins, powdery mildew resistant *T. timopheevii*, and nine *T. aestivum* (2n = 42, genome AABBDD)-*T. timopheevii* introgression lines (IGV1-465, IGV1-448, IGV1-458, IGV1-463, IGV1-464, IGV1-465, IGV1-466, IGV1-468, and IGV1-474) were kindly provided by Dr. J. Mackey, Swedish Agricultural University, Uppsala, Sweden. The sizes of introgressed 2G fragments in the above nine introgression lines have been characterized using molecular markers [17]. Three “Chinese Spring” (CS) nulli-tetrasomic lines for homoeologous group 2 were introduced from Wheat Genetics and Genomics Resources Center (WGGRC), Kansas State University, USA, and used to determine the chromosome location of *TaRLK1/2* genes. IGV1-465 and Prins were used for gene cloning of *TaRLK1/2* and its homologs, and for single-cell transient over-expression and gene-silencing, respectively. Common wheat variety Yangmai 158, which is moderately susceptible to powdery mildew, was used as the receptor of genetic transformation. A highly powdery mildew susceptible wheat variety, Sumai 3, was used for the production of fresh *Bgt* inoculums.

### *Bgt* isolates and inoculation

The naturally occurring *Bgt* population was collected from the field in Nanjing (Lat 31°14″N to 32°37″N, Lng 118°22″E to119°14″E), Jiangsu province, China, which was permitted for research. The inoculums were increased on Sumai 3 plants under a spore-proof greenhouse conditions prior to setting up the disease evaluation experiment. Inoculation was accomplished by gently shaking conidia from leaves of infected Sumai 3 plants, which was grown at 20 °C with 16 h daylight and 80 % relative humidity [19], onto the foliage of the tested lines.

### Rapid Amplified cDNA End (RACE)

The SMART^TM^ RACE cDNA Amplification Kit (Clontech, America) was used to clone the full-length cDNA of different members of the *LRR-RLK* gene family from the cDNA mixture of IGV1-465 at 1, 6, 12, 24, 36, 48 and 72 hours after inoculation (hai) according to the manufacturer’s instructions. Primers for 5’-RACE (LRR-RLK-5’ out and LRR-RLK-5’ inner) and 3’-RACE (LRR-RLK-3’) are described in Additional file 1: Table S1. Two pairs of primers (LRR-RLK1-QC-F/R and LRR-RLK2-QC-F/R) were designed based on the sequence of the 5’and 3’cDNA ends and used to obtain the full length cDNA of *TaRLK1* and *TaRLK2* (Additional file 1: Table S1). The PCR products were cloned into the pGEM-T Easy Vector (Takara, Japan) and sequenced by BGI (China).

### Sequence analysis, domain prediction, and phylogenetic analysis

Signal peptide, transmembrane and kinase domains were predicted using the SMART software (http://smart.embl-heidelberg.de/) [20]. The ATP binding site, Ser/Thr kinase active site and twin arginine translocation (Tat) signal domain were predicted by the ScanProsite software (http://prosite.expasy.org/scanprosite/).

Phylogenetic trees were constructed based on the full length amino acid sequences of TaRLK1, TaRLK2, and other LRR-RLKs. Full length of TaRLK1 and TaRLK2 were used as query sequences to identify their orthologs by the BLASTP in the Phytozome proteome database (http://www.phytozome.net/search.php?show=blast&method=Org_Cpapaya). Phylogenetic analysis was performed using MEGA 4 based on the Neighbor-Joining method and a bootstrap test of 1000 replicates [21].

### Marker analysis

Genomic DNA was extracted from young leaves as previously described [22], and the detail sequence information of the primer pairs was listed in Qin et al. [23]. PCR was performed following the procedure of Ji et al. [18] and the PCR products were separated on 8 % non-denaturing polyacrylamide gels (Acr:Bis = 19:1 or 39:1) at room temperature with 1 × TBE buffer and visualized by silver staining [24].

### Expression analysis of *TaRLK1* and *TaRLK2* by quantitative Real Time-PCR (qRT-PCR)

For *Bgt* treatments, the Prins and IGV1-465 were grown in a chamber for 16/8 h light/dark at 25/20 °C and then inoculated with *Bgt* spores onto the leaf surface at the second and fourth leaf stages. For chemical treatments, the seedlings of IGV1-465 at the first leaf stage were sprayed with 5 mM salicylic acid (SA), 100 μM methyl jasmonate (MeJA), 100 μM abscisic acid (ABA), or 7 mM hydrogen peroxide (H_2_O_2_) in 0.1 % ethanol solution, and with 0.1 % ethanol used as control. For the expression of transgenic lines, the Yangmai 158 and transgenic lines were grown in a chamber for 16/8 h light/dark at 25/20 °C. The leaves of each sample were frozen immediately in liquid nitrogen and stored at −80 °C. Total RNA was extracted using the TRIZOL reagent (Invitrogen, USA), and DNase I was used to remove the DNA before reverse transcription. The reverse transcript reaction was performed using AMV reverse transcriptase (TaKaRa, Japan) according to the manufacturer’s instructions.

qRT-PCR was performed to analyze the expression of *TaRLK1*/*TaRLK2* using SYBR Green I Master Mix (TaKaRa, Japan) in a volume of 25 μL, and the *18S rRNA* was used as a reference. The qRT-PCR reaction was performed using the ABI Prism 7000 system (Applied Biosystems, USA). The program used was as follows: 94 °C for 1 min; followed by 40 cycles of 94 °C for 5 s, 65 °C for 15 s and 72 °C for 30 s. The relative transcript level of *TaRLK1*/*TaRLK2* was calculated using the 2^–ΔΔCT^ method [25]. The sequence information of the primers (LRR-RLK-QPCR-F/R and 18S rRNA-F/R) used are listed in Additional file 1: Table S1.

### Construction of *pBI220:TaRLK* and *pWMB006:TaRLK* vectors

The ORF of *TaRLK1* and *TaRLK2*, amplified by primer pair LRR-RLK-ORF-F and LRR-RLK-ORF-R, were inserted into the expression vector *pBI220* under the control of the CaMV35S promoter, respectively. The recombinant vectors *pBI220:TaRLK1* and *pBI220:TaRLK2* were used for single-cell transient over-expression assay and genetic transformation.

The binary vector *pWMB006* (kindly provided by Dr. Xingguo Ye, Institute of Crop Science, Chinese Academy of Agricultural Sciences), which is under the control of the maize ubiquitin promoter *Ubi*, was used as an intermediate vector for RNAi vector construction [26]. The common 259 bp-fragment to both *TaRLK1* and *TaRLK2,* amplified by primer pair LRR-RLK-RNAi-F and LRR-RLK-RNAi-R (Additional file 1: Table S1), was digested with the restrictive enzymes *Spe*I and *Sac*I and then inserted into the *Spe*I-*Sac*I site in sense orientation. The fragment amplified with the same above primer pair was digested by the restrictive enzymes *BamH*I and *Kpn*I and then ligated into the *BamH*I-*Kpn*I site in antisense orientation. Accordingly, the hairpin RNAi vector *pWMB006:TaRLK* was obtained and used for single-cell transient gene-silencing assay. The linker between the two reverse fragments is a 478 bp intron from rice.

### Single-cell transient over-expression and gene-silencing assays

The single-cell transient over-expression and gene silencing assays were performed according to Shirasu et al. [27] and Douchkov et al. [28], respectively. In brief, primary leaf segments of seven-day-old wheat seedlings were transformed by tungsten particles coated with a mixture of *pAHC25* [29] containing the *GUS* gene and the recombinant vectors *pBI220:TaRLK1* or *pBI220:TaRLK2*. The bombarded wheat leaves were transferred to 1 % agar plates supplemented with 85 μM benzimidazole and incubated at 18 °C for 6 h before high density inoculation with *Bgt* spores. When RNAi vectors containing *pWMB006:TaRLK* were transformed, the bombardment leaf segments were incubated for 72 h before the inoculation with *Bgt* mixed isolates. The leaves were stained with *GUS* to observe the epidermal cells and haustorium-containing transformed cells infected by *Bgt* spores at 48 hai. The haustorium index (number of haustoria in *GUS*-expressing cells relative to the total number of *GUS*-expressing cells) was presented as the mean of three or four independent replicated experiments. Each replicate included examination of 50−200 successful *GUS*-expressing cells upon inoculation with *Bgt* conidia.

### Genetic transformation

The moderate susceptible wheat variety Yangmai 158 was used as the receptor to generate the transgenic wheat. The expression plasmid *pBI220:TaRLK1* or *pBI220:TaRLK2* was co-bombarded with the plasmid *pAHC25* into the callus from the immature embryo of Yangmai 158. Gene gun bombardment of embryos, selection, and regeneration were carried out as described by Xing et al. [30].

The genomic DNA of the regenerated T_0_ plants was isolated and used for PCR analysis using the primer pair 35S-F and LRR-RLK-R (Additional file 1: Table S1) to identify the positive transgenic plants. Semi qRT-PCR (sqRT-PCR) was performed with 30 cycles (95 °C for 1 min, 56 °C for 40 s, 72 °C for 35 s) to compare the expression of the target genes in the untransformed control and the transgenic wheat. The sequence information of the primers (LRR-RLK-RT-F and LRR-RLK-RT-R) is listed in Additional file 1: Table S1.

### Evaluation of powdery mildew resistance

Powdery mildew resistance of the transgenic line and Yangmai 158 was evaluated by artificial *Bgt* inoculation as described below.

For seedling stage resistance, the detached leaf segments of T_0_ transgenic plants and the control were maintained on the culture medium (0.5 % agar and 20 mg/l 6-BA) in a petridish, inoculated with *Bgt* isolates for 5−6 days under pathogen-free environment, and the infection types (ITs) were scored as grades 0 to 4, in which, IT 0-1 were resistant, and IT ≥ 2 were susceptible. The final results were the average of three independent experiments.

For adult stage resistance, at 6−7 days after *Bgt* inoculation of the T_0_ transgenic plants and the control in the greenhouse, their ITs were scored as grades 0 to 8 [31], in which IT 0-3 was resistant, while IT > 3 was susceptible. All the plants were scored twice.

### Histochemical staining using 3, 3’-diaminobenzidine (DAB)

*In vivo* H_2_O_2_ production in plants was detected by an endogenous peroxidase-dependent *in situ* histochemical staining procedure using DAB (Bio Basic Inc., Shanghai, China) [32]. The first detached leaves from 7-day-old seedlings after *Bgt* infection were stained with 1 mg/mL DAB dissolved in NaOH-acidified (pH 3.8) distilled water overnight, then discolored in boiling 95 % ethanol for 10 min, stored in 50 % glycerol. Representative phenotypes were captured with an Olympus microscope (MVX10, Olympus, Japan).

### Measurements of H_2_O_2_ production and antioxidant enzyme activities

The H_2_O_2_ content was measured according to Bellincampi et al. [33]. Briefly, an aliquot of supernatant (500 μL) was added to 500 μL assay reagent (500 μM ferrous ammonium sulfate, 50 mM H_2_SO_4_, 200 μM xylenol orange, 200 mM sorbitol). After 45 min of incubation, the peroxide-mediated oxidation of Fe^2+^ to Fe^3+^ was determined by measuring the absorbance at 560 nm of the Fe^3+^-xylenol orange complex.

Super oxide dismutase (SOD) and catalase (CAT) enzyme activities were analyzed following the method described by zhang et al. [34]. Total SOD activity was measured based on its ability to reduce nitroblue tetrazolium (NBT) by the superoxide anion generated by the riboflavin system under illumination. One unit of SOD (U) was defined as the amount of crude enzyme extract required to inhibit the reduction rate of NBT by 50 %. Determination of guaiacol peroxidases (POD) enzyme activity was carried out by measuring the oxidation of guaiacol extinction coefficient. CAT activity was spectro-photometrically measured by monitoring the consumption of H_2_O_2_ (extinction coefficient 39.4 mM^-1^ cm^-1^) at 240 nm for at least 3 min.

### Statistical analysis

All data obtained were subjected to ANOVA, and the mean difference was compared by the LSD test at 95 % or 99 % levels of probability. In all figures, the spread of values is shown as error bars representing standard errors of the means.

## Results

### Cloning and sequence analysis of *TaRLK1* and *TaRLK2*

Previous study suggested that a cluster of *LRR-RLK* genes located in the long arm of chromosome 2B of *T. aestivum* -*T. timopheevii* introgression line IGV1-465. In the synthetic regions of rice and *B. distachyon*, the cluster is conservely present and each has five and two *LRR-RLK* gene members*,* respectively [23]. Based on sequence of one of the two RLK gene members in *B. distachyon* (Bradi5g21860.1), two specific nest primer pairs (Additional file 1: Table S1) were designed and used for 5’ RACE (5’ RACE Inner and 5’ RACE Outer) and 3’ RACE (AUAP and AP) using cDNA of IGV1-465. The 5’- and 3’- end sequences of the *LRR-RLK* gene were obtained (Additional file 2: Table S2). According to the predicted open reading frame (ORF) of the obtained expressed sequence of a putative *LRR-RLK*, two primer pairs (LRR-RLK1-QC-F/LRR-RLK1-QC-R and LRR-RLK2-QC-F/LRR-RLK2-QC-R) were further designed and used to clone the full-length cDNA. Two members of the *LRR-RLK* gene family, namely *TaRLK1* (Genbank acc. No.: KC700615) and *TaRLK2* (Genbank acc. No.: KC700616) were obtained from IGV1-465, both containing an ORF of 3045 nucleotides encoding a 1014-amino-acid peptide.

**Fig. 1:**
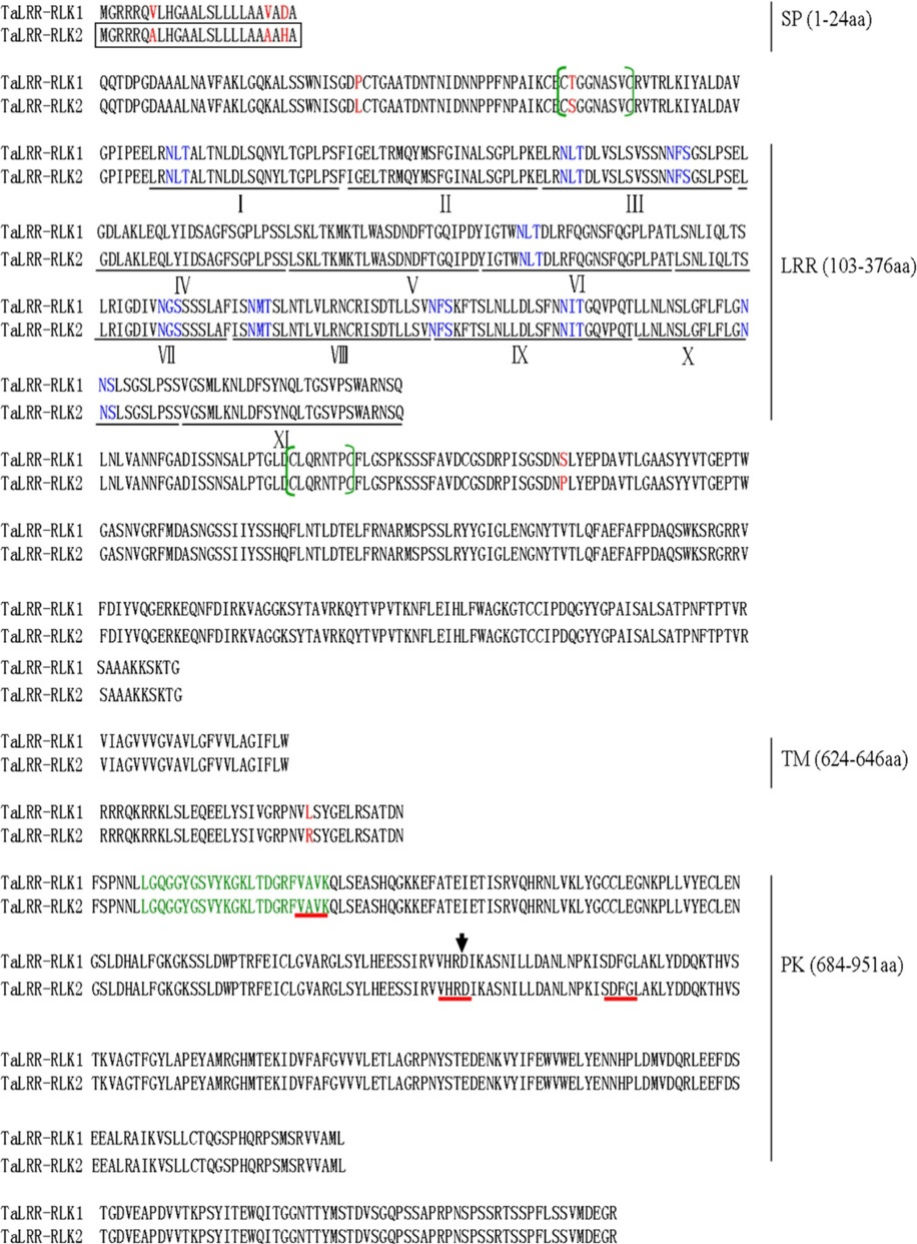
The deduced amino acid sequences of TaRLK1 and TaRLK2 proteins. Red characters represent seven amino acid differences. The open boxed region represents the N-terminal region of TaRLK2 which comprises a putative twin arginine translocation (Tat) signal domain. Characters in the green brackets represent conserved pairs of cysteines spaced by six or seven amino acids. Roman numerals mark the 11 tandem copies of a 24-amino acid LRR. Blue characters represent nine glycosylation sites (N-X-S/T). Green characters represent a putative protein kinase catalytic domain with ATP binding site. Black arrow heads indicate a Ser/Thr kinase active site (D). Characters underlined as red represent the conserved motifs (VAVK, HRD and DFG) in the RD kinases. SP: signal peptide domain; LRR: leucine-rich repeat domain; TM: transmembrane domain, PK: Ser/Thr protein kinase domain

Pair wise comparison showed that TaRLK1 and TaRLK2 share 99.31 % identity and are only different in seven amino acids, i.e. V7A, V21A, D23H, P56L, T82S, S433P and L672R (Fig. 1). The SMART software [20] predicted that both LRR-RLKs contain a putative signal peptide domain at the N-terminal region (residues 1-24), a putative extra-cellular domain (residues 103-376) with 11 tandem copies of a 24-amino acid LRR; a putative trans-membrane domain (residues 624-646) and a putative Ser/Thr protein kinase domain at the C-terminal region (residues 684-951) (Fig. 1). Each unit of the LRR domain has the LXXLXXLXXLXLXXNXLXGXLPXX consensus, in which X represents any amino acid. Based on the structural properties predicted by the ScanProsite, both LRR-RLKs consist of an ATP binding site (LGQGGYGSVYKGKLTDGRFVAVK) and a Ser/Thr kinase active site (D) in their protein kinase catalytic domains. As expected, several highly conserved motifs, Val-Ala-Val-Lys (VAVK), His-Arg-Asp (HRD), or Asp-Phe-Gly (DFG) exist in the C-terminal Arg-Asp (RD) kinase domains for both LRR-RLKs [35]. The LRR domain of both LRR-RLKs contains 9 glycosylation sites (N-X-S/T). The N-terminal region of TaRLK2 contains a putative Tat (twin arginine translocation) signal domain, which is absent in the TaRLK1 (Fig. 1).

BLASTP was performed against the Phytozome proteome database for identifying the TaRLK1/2 orthologs based on the similarity of full-length amino acid sequence. TaRLK1/2 orthologs were identified from 31 plant species with available genome information. Phylogenetic analysis revealed that the two TaRLKs and their orthologs from different *Gramineae* species are in the same branches. Both TaRLK1 and TaRLK2 are highly homologous to Bradi5g21870.2, Os04g52600, Os04g52630.1, Os04g52606.1, Os04g52640.1, Os04g52614.1, GRMZM2G126858_T02, Si009240m, and Sb06g028570.1, indicating LRR-RLK orthologs are highly conserved (Additional file 3: Figure S1, Additional file 4: Table S3).

### Physical localization of *TaRLK1*/*2*

**Fig. 2:**
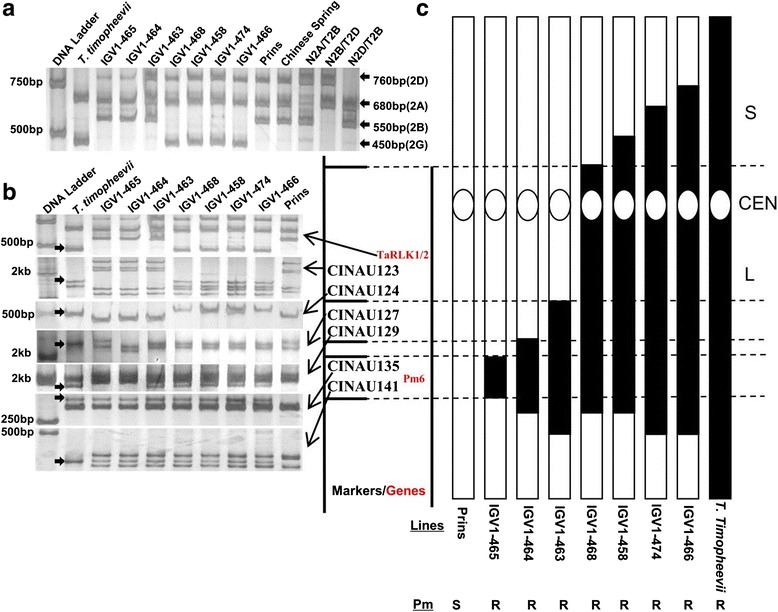
Physical localization of *TaRLK1*/*2*. PCR was conducted using specific primer pair NAU-2 F and NAU-2R differentiating *TaRLK1*/*2* on BB subgenome and its homologue genes on AA, DD and GG subgenome (**a**). The arrows point the amplified bands on 2A, 2B, 2D and 2G. *TaRLK1*/*2* homologue gene on GG subgenome was physically mapped to the same chromosome region as reported markers CINAU123 and CINAU124 (**b**) using a set of introgression lines from *T. timopheevii*.(**c**). In Fig. 2b, the arrows point the specific bands from GG subgenome. In Fig. 2c, white boxes indicate the chromosome from Prins BB subgenome and black boxes indicate introgression fragments from T. *timopheevii* GG subgenome*.* White ovals indicate centromere region. Letter S, L and CEN represent chromosome short arm, long arm and centromere. At the bottom, Pm means powdery mildew responses, resistance (R) or susceptibility (S)

In the scaffold 11030 from chromosome 2D of the released *Aegilop tauschii* (2n = 14, genome DD) genome sequence [36], a *TaRLKs* homolog (*TaRLK*-D) having highest sequence similarity, were identified. A primer pair (NAU-2 F/R) were designed and used for determine the chromosome location of *TaRLKs* from IGV1-465. NAU-2 F/R amplified three common amplicons (760 bp, 680 bp and 550 bp, respectively for chromosome 2D, 2A and 2B) in Prins and IGV1-465. A 450 bp amplicon was only present in *T. timopheevii* and four introgression lines IGV1-468, 458, 474, 466 (Fig. 2a). Six molecular markers previously mapped to the long arm of chromosome 2B [23] together with NAU-2 F/R were used for amplification in nine *T. aestivum*-*T. timopheevii* introgression lines. Based on the presence and absence of the specific amplicons for chromosome 2G, the *TaRLK1/2* could be mapped to the same chromosome region as markers CINAU123 and CINAU124, while not as markers CINAU135 and CINAU141, in which the *Pm6* is located (Fig. 2b).

Compared with the released sequence of *Triticum urartu* (Genome AA) [37], the *TaRLK1* is from the 2A, and the *TaRLK2* is from 2B. By PCR-based homologous cloning, 51, 19 and 16 full length sequences of *TaRLKs* were obtained from *Bgt* resistant IGV1-465 and *T. timopheevii* as well as the *Bgt* susceptible wheat variety Prins. Multiple sequence alignment with *TaRLK1* and *TaRLK2* identified 59 Single Nucleotide Polymorphisms (SNPs) (Table 1). Based on the distribution of the 59 SNPs, the *TaRLKs* can be classified into four different types, with the *TaRLK1 and TiRLK* were two original types. The *TaRLK2* is presumed to be originated from a recombination between *TaRLK1* and *TiRLK* at the region between 396-459 bp, and *TaRLK*_*Prins*_ is presumed to be originated from a recombination between *TaRLK1* and *TiRLK*, but at a different region (between 1054-1125 bp) (Additional file 5: Figure S2).

**Table 1:** SNPs in the *TaRLK* genes from powdery mildew resistant IGVI-465, *T. timopheevii* and powdery mildew susceptible Prins

Gene Type	SNPs									
	20	62	67	183	186	213	244	312	318	343
*TiRLK*	C	C	C	C	G	T	T	C	C	C
*TaRLK1*	T	T	T	G	C	G	A	G	A	T
*TaRLK2*	C	C	C	C	G	G	T	C	C	C
*TaRLK*_Prins_	C	C	C	C	G	G	T	C	C	C
Gene Type	SNPs									
	396	459	468	472	483	547	612	687	692	723
*TiRLK*	C	G	G	G	G	A	A	T	G	A
*TaRLK1*	T	A	A	A	A	G	G	A	C	C
*TaRLK2*	C	A	A	A	A	G	G	A	C	C
*TaRLK*_Prins_	C	G	G	G	G	A	A	T	G	A
Gene Type	SNPs									
	750	766	779	799	853	882	909	1054	1125	1128
*TiRLK*	A	G	A	C	T	T	G	C	G	A
*TaRLK1*	G	A	G	G	A	C	A	T	A	G
*TaRLK2*	G	A	G	G	A	C	A	T	A	G
*TaRLK*_Prins_	A	G	A	C	T	T	G	C	A	G
Gene Type	SNPs									
	1137	1221	1225	1248	1306	1319	1362	1404	1455	1593
*TiRLK*	G	C	T	A	C	C	G	C	A	T
*TaRLK1*	A	T	C	C	G	T	A	T	G	G
*TaRLK2*	A	T	C	C	G	T	A	T	G	G
*TaRLK*_Prins_	A	T	C	C	G	T	A	T	G	G
Gene Type	SNPs									
	1668	1847	1887	1894	1911	1917	2088	2091	2142	2146
*TiRLK*	A	T	A	A	G	G	G	G	A	C
*TaRLK1*	G	C	G	G	C	A	T	A	G	G
*TaRLK2*	G	C	G	G	C	A	T	A	G	G
*TaRLK*_Prins_	G	C	G	G	C	A	T	A	G	G
Gene Type	SNPs									
	2172	2220	2223	2276	2487	2490	2514	2517	2526	
*TiRLK*	G	G	T	A	C	C	A	C	C	
*TaRLK1*	A	C	G	G	G	T	G	T	G	
*TaRLK2*	A	C	G	G	G	T	G	T	G	
*TaRLK*_Prins_	A	C	G	G	G	T	G	T	G	

### The gene expression patterns for *TaRLK1/TaRLK2* at two leaf stages and by *Bgt* infection

*T. aestivum*-*T. timopheevii* introgression lines show high powdery mildew resistance only after the fourth leaf stage [38]. The IGV1-465 is susceptible at the first leaf stage but has a higher resistance level starting from the fourth leaf stage (Additional file 6: Figure S3) [23].

**Fig. 3:**
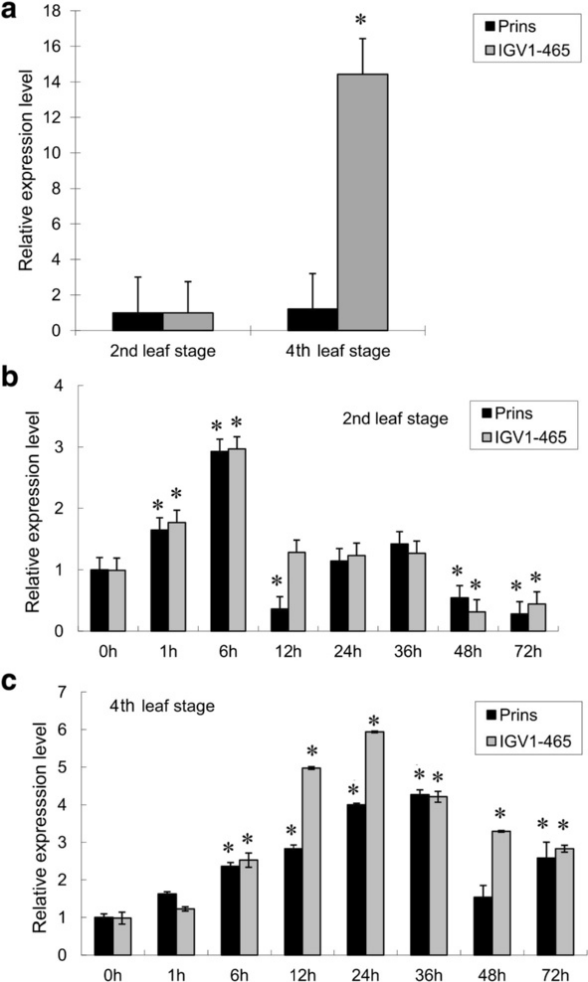
Gene expression profiling of *TaRLKs* in Prins and IGV1-465. *TaRLKs* expression in Prins and IGV1-465 at the second and fourth leaf stages without *Bgt* inoculation (**a**), and response to *Bgt* infection at the second (**b**) and fourth (**c**) leaf growing stages, respectively. “*” indicates significant differences across different time point within each genotype at 0.05 levels, using 2nd leaf stage (**a**) and non-inoculated sample (**b, c**) as control. h: hours after *Bgt* inoculation. Data were from three independently replicated experiments with similar result

The expression profiles of *TaRLK1*/*TaRLK2* in the resistant line IGV1-465 and susceptible Prins were analyzed by qRT-PCR. In IGV1-465, the expression level of *TaRLK1*/*TaRLK2* at the fourth leaf stage is 14.4 times higher than that at the second leaf stage. Whereas, in Prins, expression levels are similar for all tested time points, and remain at relatively low levels both at the second- and fourth-leaf stages (Fig. 3a). The gene expression patterns of *TaRLK1*/*TaRLK2* in IGV1-465 are consistent with the development-dependent resistance phenotype. Further comparison of the expression of *TaRLK1*/*TaRLK2* in IGV1-465 and Prins upon *Bg*t inoculation at the second and fourth leaf stages showed that, *TaRLK1*/*TaRLK2* was slightly up-regulated in both genotypes (in Prins at 6 hai: 2.9 folds; in IGV1-465 at 6 hai: 3.0 folds) at the second leaf stage (Fig. 3b). However, their expression levels were significantly up-regulated in IGV1-465 (6.0 folds at 24 hai) than those in Prins (4.0 folds at 24 hai) at the fourth leaf stage when challenged with *Bgt* infection (Fig. 3c). Above results indicated that the expression of *TaRLK1*/*TaRLK2* was development-dependent.

### Powdery mildew resistance evaluation of *TaRLKs* by transient over-expression or silencing assays

**Fig. 4:**
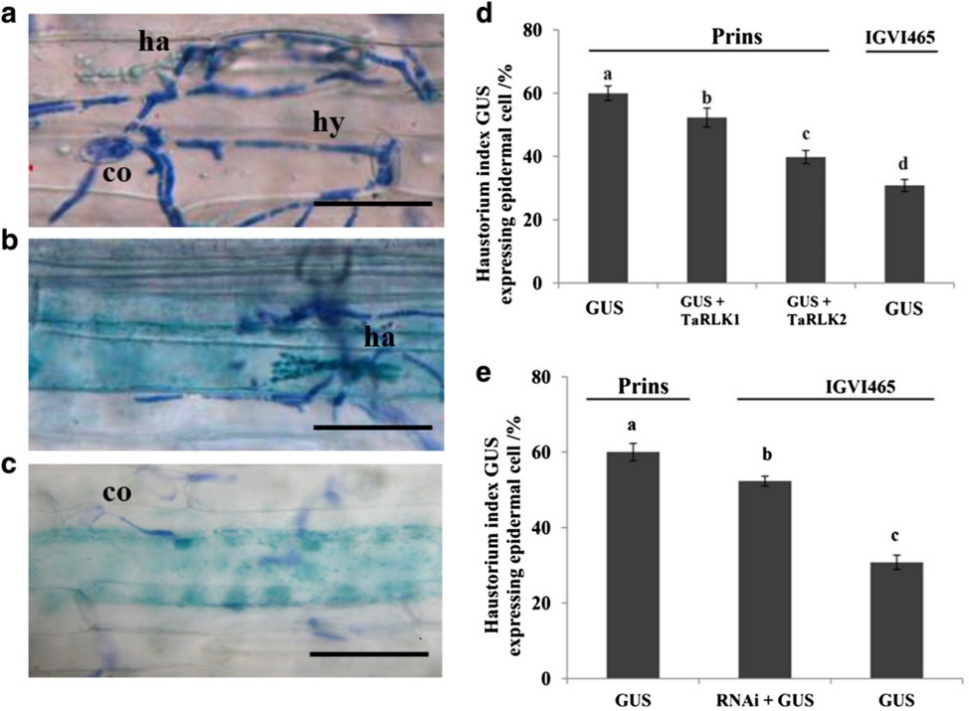
The interaction of leaf epidermal cells challenged with *Bgt* and the statistics of Haustorium Index (HI) after single-cell transient over-expression assay. Representative of compatible (**a** and **b**) and incompatible interaction (**c**) wheat leaf epidermal cells challenged with *Bgt*, the haustorium index (HI) of *Bgt* after single-cell transient expression of *TaRLK1* and *TaRLK2* in the epidermal cells in Prins (**d**) and after transient induced gene silencing of *TaRLK1/2* in IGV1-465 (**e**) co: conidia; ha: haustorium; hy: hyphae. Scale bars = 50 μm. Different letters indicate the significant difference at 0.05 levels. Data were from three independent replicated experiments

A single-cell transient over-expression assay, which has been successfully used to elucidate gene function in resistance against *Bgt* infection [9, 39], was used to elucidate the above assumption. The epidermal cells expressing reporter gene *GUS* and undergoing attacks by germinating *Bgt* spores were selected to observe the haustorium formation and calculate the Haustorium Index (HI). The interaction between the host and *Bgt* is considered compatible when haustorium and elongating secondary hyphae are formed (Fig. 4a, b). When *Bgt* fail to penetrate into the cells and no haustorium formed, the interaction is considered incompatible (Fig. 4c). Our analyses revealed that, compared with the 60.41 % HI in Prins transformed with *pAHC25* only, transient over-expression of *TaRLK1* or *TaRLK2* in leaves of Prins by co-transformation of pBI220:*TaRLK1* or pBI220:*TaRLK2* with *pAHC25* significantly decreased the HI to 53.72 % and 37.55 %, respectively.

The HI in the *Bgt* resistant line IGV1-465 transformed with *pAHC25* empty vector was 31.47 % (Fig. 4d). Whereas, when *TaRLKs* were transiently silenced in IGV1-465, the HI was significantly increased to 52.73 % (Fig. 4e). These results indicate that the *TaRLKs* positively regulates the powdery mildew resistance by suppressing *Bgt* haustorium formation.

### The function analysis of *TaRLK1* and *TaRLK2* in resistance to powdery mildew in transgenic lines

**Fig. 5:**
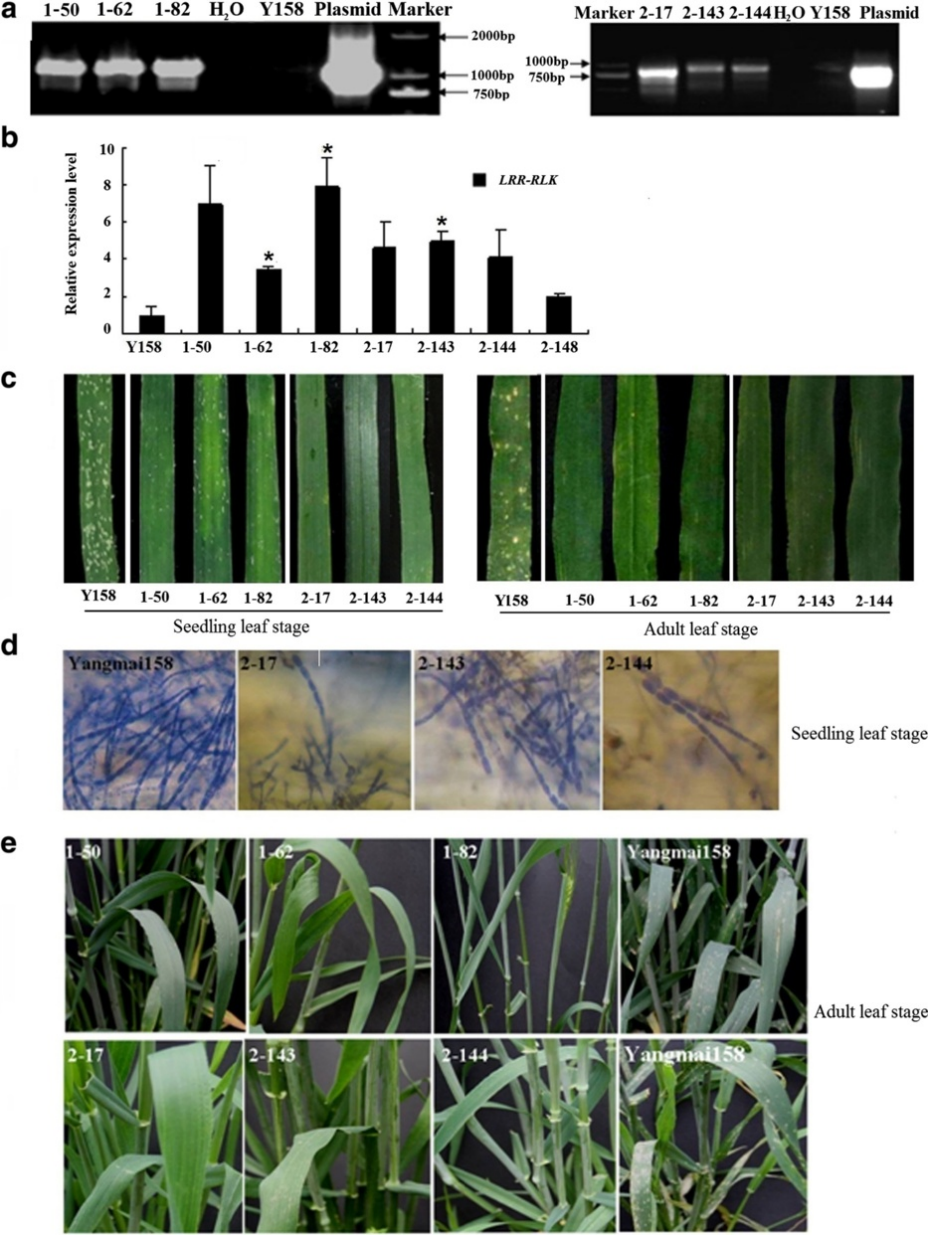
The molecular identification of *TaRLK1* and *TaRLK2* transgenic plants and powdery mildew resistance evaluation. Using specific primer pair, we performed PCR to identify *TaRLK1* and *TaRLK2* T_0_ positive transgenic plants (**a**) and qRT-PCR to study the gene expression level of *TaRLK1* and *TaRLK2* in T_0_ positive transgenic plants, “*” indicates significant differences at 0.05 levels, compared with Yangmai 158. Powdery mildew resistance evaluation for *TaRLK1* and *TaRLK2* T_0_ transgenic plants at seedling and adult leaf stage were conducted, using susceptible Yangmai 158 as control (**c**). The hyphae growing on the surface of leaves from transgenic lines for *TaRLK2* gene at seedling growing stage (**d**) and transgenic lines for *TaRLK1* and *TaRLK2* at adult growing stage (**e**) were observed, using susceptible Yangmai 158 as control

Subsequently, the *pBI220:TaRLK1* and *pBI220:TaRLK2* were each co-transformed with *pAHC25* (having a *Bar* gene encoding phosphinothric in acetyltransferase for selection) into the callus of Yangmai 158 by Genegun bombardment. After three rounds of herbicide bialaphos selection, a total of 158 and 187 regenerated plants were obtained from 2,000 and 2,240 immature embryo callus transformed *TaRLK1* and *TaRLK2*, respectively. PCR analysis using the combination primer pair CaMV35S-F and LRR-RLK-R identified 22 and 33 transgene-positive plants, respectively (Fig. 5a). The regeneration frequencies were 7.9 % (158/2,000) and 8.3 % (187/2,240), and the frequencies of transgene-positive plants were 1.1 % (22/2,000) and 1.5 % (33/2,240) for genes *TaRLK1* and *TaRLK2*, respectively. qRT-PCR analysis of part of the selected positive plants verified that the expression levels of *TaRLK1* or *TaRLK2* were higher in the transgenic plants than those in the receptor Yangmai 158 and the negative regenerated plant TaRLK2-148 (Fig. 5b).

The powdery mildew resistance of transgenic plants at T_0_ generation against *Bgt* mixture isolates was evaluated at both seedling and adult stages. At seedling stage, for example, the infection types (ITs) of three transgenic plants over-expressing *TaRLK1* (TaRLK1-50, TaRLK1-62, TaRLK1-82) were all grade 1; The ITs of three transgenic plants over-expressing *TaRLK2* (TaRLK2-17, TaRLK2-143, TaRLK2-144) were all grade 0. Whereas, the IT of non-transformed Yangmai 158 was grade 3 (Fig. 5c), indicating that the over-expression of *TaRLK1* or *TaRLK2* enhanced the powdery mildew resistance of Yangmai 158. Compared to that in Yangmai 158, three resistant transgenic plants of *TaRLK2* had fewer germinated conidia, developed hyphae and conidiophores on their leaves (Fig. 5d). At adult stage, all the above six transgenic plants showed improved powdery mildew resistance (Grade 1), compared to Yangmai 158 (Grade 8) (Fig. 5e). These indicate that both *TaRLK1* and *TaRLK2* confer powdery mildew resistance in wheat, and *TaRLK2* gene has superior effect at seedling stage.

### The involvement of SA and ROS homeostasis in the powdery mildew resistance conferred by *TaRLK1* and *TaRLK2*

Salicylic acid (SA), jasmonate (JA) and hydrogen peroxide (H_2_O_2_) are signaling molecules that regulate complex defense responses by inducing pathogenesis related (PR) genes [40]. The induction of ROS potentiates the programmed cell death (PCD). Effective defense against biotrophic pathogens is mainly due to PCD in the host, and to associated activation of defense responses regulated by the SA-dependent pathway [41].

**Fig. 6:**
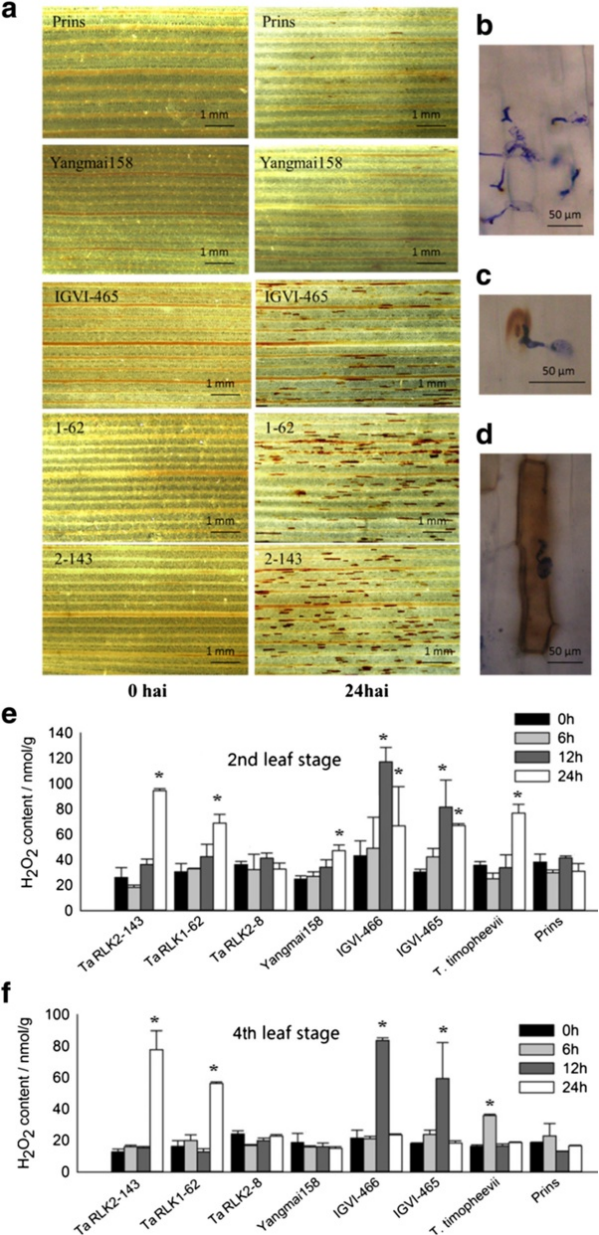
H_2_O_2_ accumulation in the leaves and endogenous H_2_O_2_ contents in different materials. Microscope observation of H_2_O_2_ accumulation in wheat leaves of Prins, Yangmai 158, IGV1-465 and the transgenic lines at 0 and 24 hai of *Bgt* (**a**) and endogenous H_2_O_2_ contents of wheat at second (**e**) and fourth (**f**) leaf stages after *Bgt* treatments. **b** was the representative image at *Bgt* interaction sites in susceptible genotypes. **c** and **d** were the representative images at *Bgt* interaction sites in resistant genotypes. H_2_O_2_ was detected at *Bgt* interaction sites in leave epidermal cells of resistant IGV1-465 and transgenic lines (**c** and **d**), while not obvious in those of susceptible Prins and Yangmai 158 (**b**). “*” indicates significant differences across different time point within each genotype at 0.05 levels, using non-inoculated sample as control. h: hours after *Bgt* inoculation. Results are replicated in three independent experiments of similar result

To elucidate the mechanism of powdery mildew resistance conferred by *TaRLK1*/*TaRLK2,* the first or second leaves from 2-week-old plants of Prins, IGV1-465, Yangmai 158 and the *TaRLK1*/*TaRLK2* transgenic plants at the T_1_ generation were DAB-stained to investigate H_2_O_2_ accumulation upon *Bgt* infection. At 0 hai, no oxidative production was observed in the leaves of any tested samples (Fig. 6a). At 24 hai, IGV1-465 exhibited higher ROS level than in Prins. The resistant transgenic lines TaRLK1-62 and TaRLK2-143 also showed more ROS than Yangmai 158 (Fig. 6a). At most of the *Bgt* interaction sites, no oxidative production was detected in Prins and Yangmai 158 (Fig. 6b). However, in TaRLK1-62, TaRLK2-143, and IGV1-465, massive H_2_O_2_ accumulation was observed at either the *Bgt* interaction sites (Fig. 6c) or in the whole cells (Fig. 6d). Time course tests of endogenous H_2_O_2_ levels showed that, at both seedling and adult stages, there was a fast endogenous H_2_O_2_ production in the two positive transgenic lines (24 hai), IGV1-465 (12 hai), IGV1-466 (12 hai) and *T. timopheevii* (12 hai and 6 hai at the second and fourth leaf stages, respectively) upon *Bgt* infection. No significant change was observed in the susceptible lines (Fig. 6e, f), such as Yangmai 158, Prins and another negative transgenic plant TaRLK2-8 which was an alternative of TaRLK2-148. These suggested that ROS pathway participated in the powdery mildew resistance conferred by *TaRLKs*.

The activities of enzymes responsible for H_2_O_2_ production and ROS scavenging were further tested. In response to *Bgt* inoculation, the H_2_O_2_ producing SOD enzyme activities significantly increased in the powdery mildew resistant materials, although different genotypes showed different patterns. At the second leaf stage, the SOD activities in the transgenic lines of both *TaRLKs* increased and reached the peak at 24 hai, while at the fourth leaf stage, significant increase advanced to 6 hai. In IGV1-465 and IGV1-466, the SOD activity increase was observed at 12 hai at the second leaf stage; however, no significant change was observed at the fourth leaf stage. In *T. timopheevii,* a significant increase of SOD activity was only observed at the fourth leaf stage. However, both Yangmai 158 and the negative transgenic line TaRLK2-8 showed significant decrease of SOD activities at 24 hai and 6 hai, respectively (Additional file 7: Figure S4).

In response to *Bgt* inoculation at two developmental stages, the activities of ROS scavenging enzymes CAT and POD increased significantly in all the resistant materials, but decreased in Yangmai 158 and Prins. In the transgenic lines of both *TaRLKs*, the increased POD activity occurred at 24 hai at the second leaf stage, and advanced to 12 hai at the fourth leaf stage; the increase of CAT activity occurred at 24 hai and 12 hai at the second leaf stages respectively in *TaRLK1* and *TaRLK2*, but advanced to 6 hai at the fourth leaf stage. In *T. timopheevii*, the POD activity increase occurred earlier than CAT (Additional file 7: Figure S4). The distinct difference of ROS production and scavenging enzymes in the resistant and susceptible materials at different developmental stages further support the importance of ROS in powdery mildew resistance mediated by the two *TaRLKs*.

**Fig. 7:**
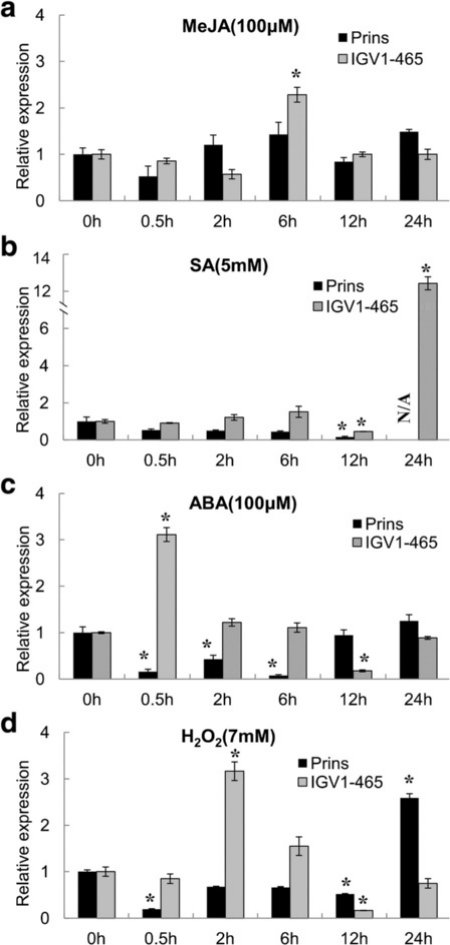
Gene expression patterns for *TaRLK1/2* in response to the treatments of exogenous phytohormones MeJA (**a**), SA (**b**), ABA (**c**) and signal molecule H_2_O_2_ (**d**) in Prins and IVG1-465. “*” indicates significant differences across different time point within each genotype at 0.05 levels, using non-inoculated sample as control. h: hours after treatment. N/A: data missing

Gene expression patterns for *TaRLK1*/*2* in Prins and IGV1-465 when treated with SA, MeJA, ABA and H_2_O_2_ were compared. In IGV1-465, *TaRLK1*/*TaRLK2* was moderately up-regulated by MeJA (2.4 folds at 6 hai, Fig. 7a), ABA (3.1 folds at 0.5 hai, Fig. 7c) and H_2_O_2_ (3.2 folds at 2 hai, Fig. 7d), and was dramatically induced by SA (13.4 folds higher, Fig. 7b) at 24 hai. While in Prins, no increased expression was detected, except when treated with H_2_O_2_, a delayed induction at 24 hai was observed (Fig. 7d). We also found that the expression of *TaPR1*, *TaPR2* and *TaPR3,* three marker genes of SA signaling pathway, was constitutively expressed at the seedling stage without *Bgt* inoculation in the transgenic lines over-expressing *TaRLKs*. The expression of *CAT*, scavenger of ROS, was up-expressed in *TaRLK1*/*TaRLK2* transgenic lines without *Bgt* inoculation (Additional file 8: Figure S5). These indicate multiple signal pathways may influence the resistance conferred by *TaRLK1*/*TaRLK2* in wheat.

## Discussion

Plant LRR-RLKs, for example, *Arabidopsis* FLS2 and rice Xa21, were found to be involved in plant immune responses to the microbe attacks [4, 42]. Both TaRLK1 and TaRLK2 contain signal peptide, LRR, TM, S/TPK domain, and several N-glycosylation sites, which have also been identified in the LRR-containing ectodomain of cell-surface receptors that can recognize MAMPs to activate PTI response in both animals and plants [43–45]. *Arabidopsis* defense-associated LRR-RLKs, such as brassinosteroid insensitive 1-associated receptor kinase [46], Flg22-induced receptor-like kinase 1 (FRK1) [47], and the PEPtide 1 and PEPtide 2 Receptors [48], are all RD kinases. Similar to *Arabidopsis* RKF1, which is responsible for triggering a cascade of intracellular events during defense responses [49], TaRLK1 and TaRLK2 containing the N-glycosylation sites and RD motif belong to the VIII-2 subfamily due to their high homology to Bradi5g21870.2, which is a member of the VIII-2 subfamily in *Brachypodium* (http://www.brachypodium.org/).

Both genes contributed to powdery mildew resistance, but showed distinct resistance level. The transgenic plants over-expressing *TaRLK1* were moderately resistant at seedling stage, and highly resistant at adult stage, while transgenic plants for *TaRLK2* exhibited high resistance at both stages, implying that both *TaRLKs* contributed to the defense response to *Bgt* infection, and *TaRLK2* had larger effects than *TaRLK1*. The Tat domain of LRR-RLKs was reported to function in transporting folded proteins across energy-transducing membranes [50]. Therefore, we assume that differential resistance may be due to their sequence differentiation at the Tat signal domain and spacer region of same gene family, which driven by co-evolution with the pathogen. The two genes had different resistance spectrums, so their different resistance levels to *Bgt* mixture may also be due to the composition of the *Bgt* isolates.

Many disease resistance genes belong to multi-gene families, indicating that gene duplication and subsequent diversification are common for gene evolution in plant [51, 52]. Recombination at the disease resistance loci, such as the disease resistance gene *Rp1* in maize and wheat stem rust resistance gene *Sr33* has been proven to be associated with the creation of novel resistance phenotypes [53, 54] and contribute to the diversification of plant gene families. Our results suggest a model for the evolution of the *TaRLK* gene family. Duplication and subsequent divergence of a progenitor *TaRLK* gene led to the emergence of *TaRLK* multi-gene families. The *TaRLK2* and *TaRLK*_*Prins*_ were originated from the types of sequence recombination between *TaRLK1* and *TiRLK1*. The association of the gene structure with their function diversification will be further elucidated.

*Pm6* was introgressed from *T. timopheevii* into common wheat [15]. *Pm6* mediated powdery mildew resistance showed a developmentally-dependence, and has been widely used in wheat breeding programs. IGV1-465 show high resistance to the local *Bgt* population, starting from the fourth leaf stage [23]. In this paper, we observed that the expression of *TaRLK1/TaRLK2* in IGV1-465 at the fourth leaf stage was higher than that at the second leaf stage (Fig. 3a). Over-expression of *TaRLK1* in Yangmai 158 via stable transformation resulted in moderately higher powdery mildew resistance at both seedling and adult stages, and over-expression of *TaRLK2* in Yangmai 158 significantly enhanced resistance at both stages (Fig. 5c, d, and e). According to these findings, we infer that both *TaRLKs* were regulated at two different stages. *Pm6* was previously mapped on chromosome 2BL within the fraction length (FL) 0.50-1.00 [16–18], flanked by two STS markers, NAU/STSBCD135-1 and NAU/STSBCD135-2, with a genetic distance of 0.8 cM [18]. In the present study the *TaRLK1*/*TaRLK2* was mapped to homeologous group 2 chromosomes of *T. aestivum* (Fig. 2a). However, we found that the specific amplicon for *TaRLK1*/*TaRLK2* from the GG genome was only present in IGV1-466 and not in IGV1-465 (Fig. 2a). IGV1-465 has the smallest introgression fragment of 2G, while IGV1-466 has the largest [17]. The absence of the 2G specific amplicon (450 bp in Fig. 2a) reveal that *TaRLK1*/*TaRLK2* were not located in the 2G chromosome introgression fragment in IGV1-465. However, they have clear function in powdery mildew resistance in wheat, and we speculated that *TaRLKs* are new powdery mildew resistance genes neighboring with the *Pm6*.

We observed that when challenged with *Bgt*, plants over-expressing *TaRLK1/2* showed enhanced H_2_O_2_ accumulation at as early as 12 hai (Fig. 6a). The SOD, POD and CAT activities increased significantly in resistant lines (IGV1-465 and IGV1-466), as well as *TaRLK1/2* over-expressing plants, mostly at 24 hai upon *Bgt* inoculation (Additional file 7: Figure S4), suggesting that the increased H_2_O_2_ production could in turn trigger the activity of ROS-scavenging enzymes, to maintain the appropriate levels of endogenous ROS. ROS, superoxide, hydrogen peroxide and nitric oxide, are produced at all levels in resistance reactions in plants. In basal resistance, they are linked to papilla formation and the assembly of barriers. In the R gene mediated defense, they may be linked to PCD, and resulted in systemic acquired resistance (SAR). They interact with SA in signaling, which is the typical pathway against a biotroph pathogen in *Arabidopsis* [41]. At least two distinct enzymes, POD and CAT, contribute to the removal of ROS [55, 56]. The HR is accompanied by a localized increase in the accumulation of ROS and is further characterized by rapid PCD at sites of infection [57]. Our data affirmed the association of *TaRLK1/2* mediated powdery mildew resistance with the ROS homeostasis, which has been proven to be responsible for triggering defense response in plants [58].

The expression of *TaRLK1*/*TaRLK2* was up-regulated significantly by SA. Moreover, the marker genes of the SA signaling pathway, *TaPR1* and *TaPR2*, were all constitutively up-regulated at the seedling stage without *Bgt* inoculation in the positive T_0_ transgenic plants over-expressing *TaRLK1* or *TaRLK2* (Additional file 8: Figure S5), indicating that both genes were involved in the SA-mediated defense pathway against *Bgt*. This implies the predominant role of the SA pathway in the *TaRLK1* or *TaRLK2* mediated powdery mildew resistance. The marker gene of ETH pathway, *PR3*, was also significantly up-regulated in the transgenic plants, indicating the possible involvement of ETH signaling pathway in the *TaRLK1* or *TaRLK2*- mediated powdery mildew resistance.

## Conclusions

In conclusion, we cloned two members of *TaRLK* family, named *TaRLK1* and *TaRLK2*, from *T. aestivum* c.v. Prins-*T. timopheevii* introgression line IGV1-465. The two genes are present as a gene cluster on the long arm of chromosome 2B, and both *TaRLK1* and *TaRLK2* confer powdery mildew resistance, which was proved by single-cell transient over-expression, gene-silencing assays and stable genetic transformation. SA and altered ROS homeostasis are involved in defense responses of the transgenic wheat to *Bgt* infection.

## Availability of supporting data

The data sets supporting the results of this article are included within the article and its additional files.

## Additional files

Additional file 1: Table S1. Sequence information of primer pairs used in this study. (DOC 48 kb) Additional file 2: Table S2. Biochemical characteristics of *TaRLK1* and *TaRLK2. (DOC 27 kb)*
Additional file 3: Figure S1. Phylogenetic analysis of TaRLK1 and TaRLK2 with other LRR-RLKs *Sorghum bicolor* (Sb06g028570.1); *Zea mays* (GRMZM2G126858_T02); *Setaria italic* (Si009240m); *Oryza sativa* (LOC_Os04g52600.1, LOC_Os04g52640.1, LOC_Os04g52630.1, LOC_Os04g52614.1, LOC_Os04g52606.1); *Brachypodium distachyon* (Bradi5g21870.2 ); *Arabidopsis thaliana* (AT1G56130.1); *Manihot esculenta* (cassava4.1_001407m); *Ricinus communis* (30169.m006328); *Linum usitatissimum* (Lus10031199); *Populus trichocarpa* (POPTR_0007s08160.1); *Cucumis sativus* (Cucsa.239700.1); *Prunus persica* (ppa017049m); *Malus domestica* (MDP0000207688); *Arabidopsis lyrata* (924153); *Capsella rubella* (Carubv10012587m); *Brassica rapa* (Bra030813); *Thellungiella halophila* (Thhalv10018064m); *Carica papaya* (evm.model.supercontig_748.4); *Citrus sinensis* (orange1.1g001658m); *Citrus clementina* (Ciclev10007054m); *Eucalyptus grandis* (Eucgr.F02391.1); *Vitis vinifera* (GSVIVT01029720001); *Mimulus guttatus* (mgv1a000612m); *Aquilegia coerulea* (Aquca_030_00368.1); *Selaginella moellendorffii* (77447); *Physcomitrella patens* (Pp1s244_27V6.2); *Panicum virgatum* (Pavirv00031150m); *Medicago truncatula* (Medtr5g091950.1); *Glycine max* (Glyma08g25590.2); *Phaseolus vulgaris* (Phvul.011G169300.1). (DOC 1203 kb) Additional file 4: Table S3. Origin, accession number and sequence comparison of *TaRLK1*, *TaRLK2* and their orthologs. (DOC 68 kb) Additional file 5: Figure S2. The schematic diagram of different types of *TaRLKs*. The *TaRLK2* was presumed to originate from a recombination between *TaRLK1* and *TiRLK* at the region between 396-459 bp. *TaRLK*_*Prins*_ was also presumed to be originated from a recombination between *TaRLK1* and *TiRLK*, but at a different region (between 1054-1125 bp). (DOC 121 kb) Additional file 6: Figure S3. Powdery mildew resistance to *Bgt* isolates mixture of Prins, IGVI-465 and Yangmai158 at the first leaf stage. (DOC 761 kb) Additional file 7: Figure S4. The detection of SOD, POD and CAT activities in transgenic plants at the second (left) and fourth (right) leaf growing stages after *Bgt* treatments. * indicates significant difference within each genotypes at 0.05 levels, using non-inoculated samples as control. The data were from three replicated independent experiments. (DOC 2439 kb) Additional file 8: Figure S5. Expression of four defense-related marker genes in the wild-type Yangmai 158 and *TaRLK1*/*TaRLK2* transgenic plants without the inoculation of *Bgt* (* *p* < 0.05, ** *p* < 0.01). (DOC 126 kb)

## Abbreviations

*Bgt*: *Blumeria graminearum f.sp tritici*
ROS: reactive oxygen species
SA: salicylic acid
H_2_O_2_: hydrogen peroxide
JA: jasmonate acid
ABA: abscisic acid
RLK: receptor-like kinase
PRRs: pattern recognition receptors
PAMPs: pathogen-associated molecular patterns
PTI: pattern triggered immunity
FLS2: Flagellin Sensitive 2
EFR: EF-Tu receptor
LysM: lysine-motif
LecRK: lectin receptor kinase
AtCERK1: chitin elicitor receptor kinase 1 of *Arabidopsis*
CS: Chinese Spring
WGGRC: Wheat Genetics and Genomics Resources Center
Tat: twin arginine translocation
ITs: infection types
DAB: diaminobenzidine
SOD: super oxide dismutase
CAT: catalase
NBT: nitroblue tetrazolium
POD: guaiacol peroxidases
SNPs: Single Nucleotide Polymorphisms
HI: Haustorium Index
PCD: programmed cell death
FRK1: Flg22-induced receptor-like kinase 1
