# Supplementary material for: Two members of TaRLK family confer powdery mildew resistance in common wheat
Source: BMC Plant Biol. 2016 Jan 25;16:27. doi: 10.1186/s12870-016-0713-8 (PMC4727334; doi:10.1186/s12870-016-0713-8)
Supplement: Additional file 1: Table S1. — Sequence information of primer pairs used in this study. (DOC 48 kb) [file 12870_2016_713_MOESM1_ESM.doc]

**Additional file 1: Table S1.** Sequence information of primer pairs used in this study

| **Name of the primers** | **Primer sequences (5**′**-3**′**)** | **Used for** |
| --- | --- | --- |
| LRR-RLK -5′out | CCAGAAGAGATGAATCTCAAGAAAG | Primers for RACE |
| LRR-RLK-5′inner | CCTTTCTTATGTCAAAGTTCTGCTC |
| LRR-RLK -3′ | ATATATATGTCCAGGGTGAGCGTAA |
| LRR-RLK1-QC-F | ATGGGGGCTCACCAGCTCTCTTATT | Primers for full length amplification of *TaLRR-RLK1* |
| LRR-RLK1-QC-R | GAGACCAAAGAATTCAGAGCTTC |
| LRR-RLK2-QC-F | TAGAGCACCAGGCAGATAGCCA | Primers for full length amplification of *TaLRR-RLK2* |
| LRR-RLK2-QC-R | ACTGCAAATCGTACGCGCGTCCAA |
| LRR-RLK-QPCR-F | TGCTGCATTCCTGATCAAGG | Primers for qRT-PCR of *TaLRR-RLK* |
| LRR-RLK-QPCR-R | GGTGAAATTTGGTGTTGCAC |
| LRR-RLK-ORF-F | CGGGATCCATGGGGCGGAGGAGGCA | Primers for ORF amplification |
| LRR-RLK-ORF-R | GGGGTACCTCAACGGCCTTCGTCCA |
| LRR-RLK-RNAi-F | CGGGGTACCACTAGTGATTTCGGGCTG | Primers for RNAi vector construction |
| LRR-RLK-RNAi-R | CGGGATCCGAGCTCCCACCATATCCAA |
| CaMV35S -F | AGTTCATTTCATTTGGAGAGAACAC | Primers for detection in the transgenic plants |
| LRR-RLK-R | ATAGATCCAAATTGGTCAAGGCCG |
| TaPR1-F | GAGAATGCAGACGCCCAAGC | Primers for *TaPR1* |
| TaPR1-R | CTGGAGCTTGCAGTCGTTGATC |
| TaPR2-F | GCAGCTCTACAGGTCCAAGG | Primers for *TaPR2* |
| TaPR2-R | CGGCGATGTACTTGATGTTG |
| TaPR3-F | ACCTCCTTGGCGTCAGCT | Primers for *TaPR3* |
| TaPR3-R | TCGCACCATTATTCCCTT |
| TaCAT-F | TGCCTGTGTTTTTTATCCGAGA | Primers for *TaCAT* |
| TaCAT-R | CTGCTGATTAAGGTGTAGGTGTT |
| LRR-RLK-RT-F | TTGGCCTTGAAAATGGAAAC | Primers for sqRT-PCR of *TaLRR-RLK* |
| LRR-RLK-RT-R | GCCACGAACCTACCATCAGT |
| 18S rRNA -F | AACACTTCACCGGACCATTCA | Primers for the internal control gene |
| 18S rRNA -R | CGTCCCTGCCCTTTGTACAC |
| NAU-2F | GCGTTATCTGGACCTTTGCC | Primers for location of *TaLRR-RLK* gene family |
| NAU-2R | GGAAGCGAGCCACTGAAATT |
