# Supplementary material for: Two members of TaRLK family confer powdery mildew resistance in common wheat
Source: BMC Plant Biol. 2016 Jan 25;16:27. doi: 10.1186/s12870-016-0713-8 (PMC4727334; doi:10.1186/s12870-016-0713-8)
Supplement: Additional file 2: Table S2. — Biochemical characteristics of TaRLK1 and TaRLK2. (DOC 27 kb) [file 12870_2016_713_MOESM2_ESM.doc]

**Additional file 2: Table S2.** Biochemical characteristics of TaRLK1 and TaRLK2

|  | 5′-UTR(bp) | 3′-UTR(bp) | MW(kDa) | PI |
| --- | --- | --- | --- | --- |
| TaRLK1 | 87 | 250 | 110.38 | 110.4 |
| TaRLK2 | 44 | 221 | 6.67 | 7.16 |
