# Supplementary material for: Two members of TaRLK family confer powdery mildew resistance in common wheat
Source: BMC Plant Biol. 2016 Jan 25;16:27. doi: 10.1186/s12870-016-0713-8 (PMC4727334; doi:10.1186/s12870-016-0713-8)
Supplement: Additional file 3: Figure S1. — Phylogenetic analysis of TaRLK1 and TaRLK2 with other LRR-RLKs Sorghum bicolor (Sb06g028570.1); Zea mays (GRMZM2G126858_T02); Setaria italic (Si009240m); Oryza sativa (LOC_Os04g52600.1, LOC_Os04g52640.1, LOC_Os04g52630.1, LOC_Os04g52614.1, LOC_Os04g52606.1); Brachypodium distachyon (Bradi5g21870.2 ); Arabidopsis thaliana (AT1G56130.1); Manihot esculenta (cassava4.1_001407m); Ricinus communis (30169.m006328); Linum usitatissimum (Lus10031199); Populus trichocarpa (POPTR_0007s08160.1); Cucumis sativus (Cucsa.239700.1); Prunus persica (ppa017049m); Malus domestica (MDP0000207688); Arabidopsis lyrata (924153); Capsella rubella (Carubv10012587m); Brassica rapa (Bra030813); Thellungiella halophila (Thhalv10018064m); Carica papaya (evm.model.supercontig_748.4); Citrus sinensis (orange1.1g001658m); Citrus clementina (Ciclev10007054m); Eucalyptus grandis (Eucgr.F02391.1); Vitis vinifera (GSVIVT01029720001); Mimulus guttatus (mgv1a000612m); Aquilegia coerulea (Aquca_030_00368.1); Selaginella moellendorffii (77447); Physcomitrella patens (Pp1s244_27V6.2); Panicum virgatum (Pavirv00031150m); Medicago truncatula (Medtr5g091950.1); Glycine max (Glyma08g25590.2); Phaseolus vulgaris (Phvul.011G169300.1). (DOC 1203 kb) [file 12870_2016_713_MOESM3_ESM.doc]

**Additional file 3: Figure S1.**

**
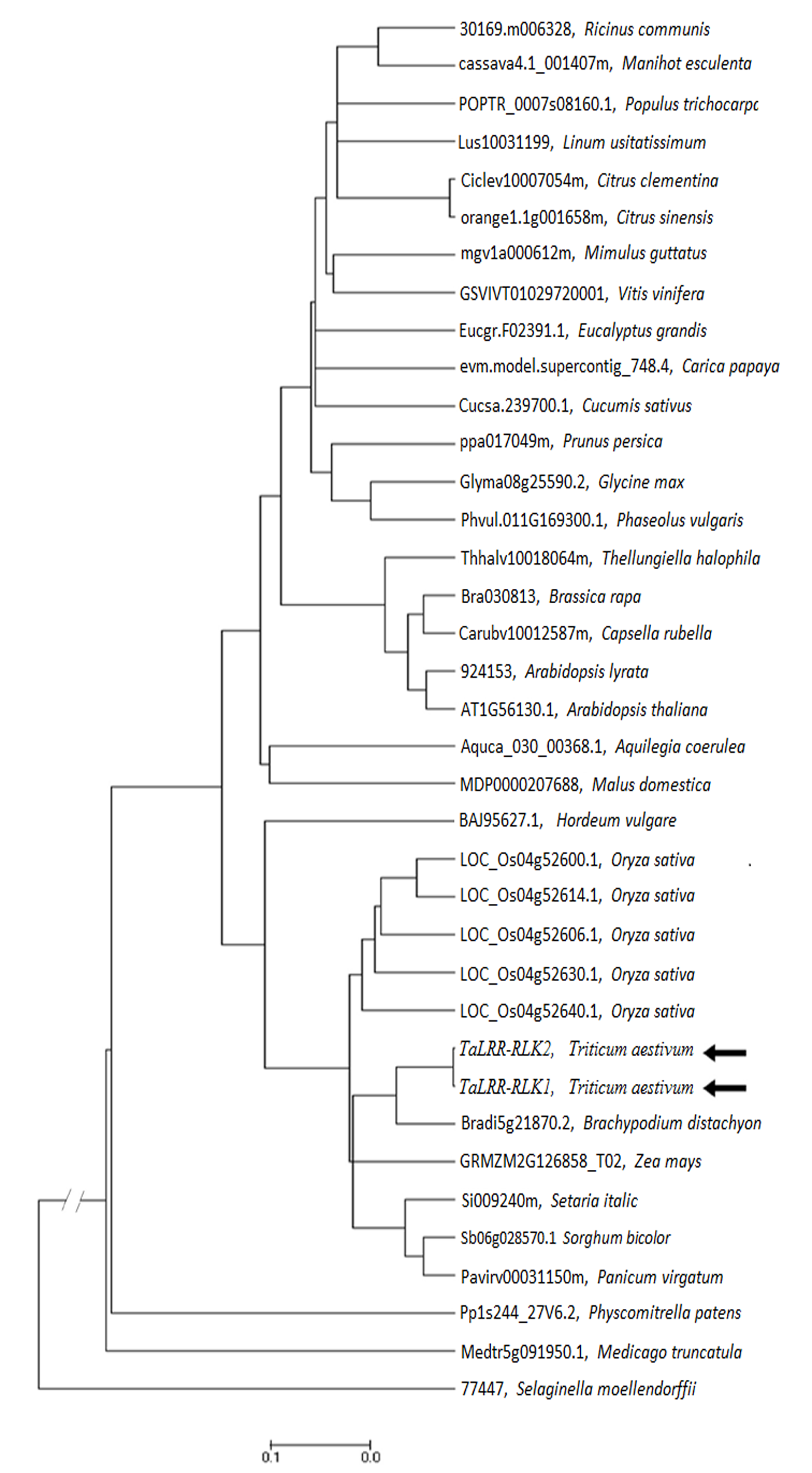
**

**Additional file 1: Figure S1.** Phylogenetic analyses of TaRLK1 and TaRLK2 with other LRR-RLKs

*Sorghum bicolor* (Sb06g028570.1); *Zea mays* (GRMZM2G126858_T02); *Setaria italic* (Si009240m); *Oryza sativa* (LOC_Os04g52600.1, LOC_Os04g52640.1, LOC_Os04g52630.1, LOC_Os04g52614.1, LOC_Os04g52606.1); *Brachypodium distachyon* (Bradi5g21870.2 ); *Arabidopsis thaliana* (AT1G56130.1); *Manihot esculenta* (cassava4.1_001407m); *Ricinus communis* (30169.m006328); *Linum usitatissimum* (Lus10031199); *Populus trichocarpa* (POPTR_0007s08160.1); *Cucumis sativus* (Cucsa.239700.1); *Prunus persica* (ppa017049m); *Malus domestica* (MDP0000207688); *Arabidopsis lyrata* (924153); *Capsella rubella* (Carubv10012587m); *Brassica rapa* (Bra030813); *Thellungiella halophila* (Thhalv10018064m); *Carica papaya* (evm.model.supercontig_748.4); *Citrus sinensis* (orange1.1g001658m); *Citrus clementina* (Ciclev10007054m); *Eucalyptus grandis* (Eucgr.F02391.1); *Vitis vinifera* (GSVIVT01029720001); *Mimulus guttatus* (mgv1a000612m); *Aquilegia coerulea* (Aquca_030_00368.1); *Selaginella moellendorffii* (77447); *Physcomitrella patens* (Pp1s244_27V6.2); *Panicum virgatum* (Pavirv00031150m); *Medicago truncatula* (Medtr5g091950.1); *Glycine max* (Glyma08g25590.2); *Phaseolus vulgaris* (Phvul.011G169300.1).
