# Supplementary material for: Two members of TaRLK family confer powdery mildew resistance in common wheat
Source: BMC Plant Biol. 2016 Jan 25;16:27. doi: 10.1186/s12870-016-0713-8 (PMC4727334; doi:10.1186/s12870-016-0713-8)
Supplement: Additional file 4: Table S3. — Origin, accession number and sequence comparison of TaRLK1, TaRLK2 and their orthologs. (DOC 68 kb) [file 12870_2016_713_MOESM4_ESM.doc]

**Additional file 4: Table S3. Origin, accession number and sequence comparison of *TaRLK1*, *TaRLK2* and their orthologs**

| **Name of the Species** | **Accession number or locus name** | **TaRLK1** | | **TaRLK2** | |
| --- | --- | --- | --- | --- | --- |
|  |  | **Identities** | **Positive** | **Identities** | **Positive** |
| *Hordeum vulgare* | BAJ95627.1 | 607/991(61%) | 749/991(75%) | 61% (606/991) | 75%(747/991) |
| *Sorghum bicolor* | Sb06g028570.1 | 71.1%(691/970) | 79.5%(771/970) | 71.0%(689/970) | 79.3%(769/970) |
| *Zea mays* | GRMZM2G126858_T02 | 65.3%(618/947) | 76.1%(721/947) | 65.2%(617/947) | 75.9%(719/947) |
| *Setaria italica* | Si009240m | 80.9%(783/968) | 89.5%(866/968) | 80.7%(781/968) | 89.2%(863/968) |
| *Oryza sativa* | LOC_Os04g52600.1 | 77.3%(751/972) | 86.1%(837/972) | 77.1%(749/972) | 86.0%(836/972) |
|  | LOC_Os04g52640.1 | 75.4%(718/952) | 85.2%(811/952) | 75.3%(717/952) | 85.0%(809/952) |
|  | LOC_Os04g52630.1 | 68.7%(666/969) | 78.6%(762/969) | 68.5%(664/969) | 78.5%(761/969) |
|  | LOC_Os04g52614.1 | 66.9%(662/989) | 75.8%(750/989) | 66.7%(660/989) | 75.7%(749/989) |
|  | LOC_Os04g52606.1 | 75.6%(456/603) | 85.1%(513/603) | 75.6%(456/603) | 84.9%(512/603) |
| *Brachypodium distachyon* | Bradi5g21870.2 | 82.5%（796/965） | 88.5% (854/965) | 82.5%(796/965) | 88.4% (853/965) |
| *Arabidopsis thaliana* | AT1G56130.1 | 55.2%（526/953） | 70.9%(676/953) | 55.4%(528/953) | 71.0%(677/953) |
| *Manihot esculenta* | cassava4.1_001407m | 60.2% (510/847) | 74.9% (634/847) | 60.2%(510/847) | 74.9%(634/847) |
| *Ricinus communis* | 30169.m006328 | 58.4%(562/963) | 73.6%(709/963) | 58.6%(564/963) | 73.7%(710/963) |
| *Linum usitatissimum* | Lus10031199 | 56.0% (546/975) | 71.5% (697/975) | 56.1%(547/975) | 71.7%(699/975) |
| *Populus trichocarpa* | POPTR_0007s08160.1 | 57.9%(560/968) | 73.2%(709/968) | 57.9%(560/968) | 73.3%(710/968) |
| *Cucumis sativus* | Cucsa.239700.1 | 58.7% (559/953) | 73.3% (699/953) | 58.9%(561/953) | 73.5%(700/953) |
| *Prunus persica* | ppa017049m | 57.5% (557/969) | 72.9% (706/969) | 57.5%(557/969) | 72.8%(705/969) |
| *Malus domestica* | MDP0000207688 | 55.3% (446/807) | 69.8% (563/807) | 55.3%(446/807) | 69.8%(563/807) |
| *Arabidopsis lyrata* | 924153 (scaffold_105434.1) | 56.0% (534/953) | 72.0% (686/953) | 56.2%(536/953) | 72.0%(686/953) |
| *Capsella rubella* | Carubv10012587m | 55.9% (530/948) | 71.3% (676/948) | 55.9%(530/948) | 71.4%(677/948) |
| *Brassica rapa* | Bra030813 | 54.8% (526/959) | 70.5% (676/959) | 54.8%(526/959) | 70.5%(676/959) |
| *Thellungiella halophila* | Thhalv10018064m | 55.7% (535/960) | 70.4% (676/960) | 55.8%(536/960) | 70.5%(677/960) |
| *Carica papaya* | evm.model.supercontig_748.4 | 59.8% (326/545) | 76.0% (414/545) | 59.8% (326/545) | 76.0% (414/545) |
| *Citrus sinensis* | orange1.1g001658m | 57.9% (558/964) | 72.8% (702/964) | 57.9%(558/964) | 72.7%701/964() |
| Continued |  |  |  |  |  |
|  |  |  |  |  |  |
| *Citrus clementina* | Ciclev10007054m | 57.2% (536/937) | 71.5% (670/937) | 57.2%(536/937) | 71.4%(669/937) |
| *Eucalyptus grandis* | Eucgr.F02391.1 | 56.2% (542/964) | 72.7% (701/964) | 56.2%(542/964) | 72.6%(700/964) |
| *Vitis vinifera* | GSVIVT01029720001 | 59.2% (577/974) | 73.1% (712/974) | 59.2%(577/974) | 73.0%(711/974) |
| *Mimulus guttatus* | mgv1a000612m | 57.4% (561/978) | 72.2% (706/978) | 57.4%(561/978) | 72.2%(706/978) |
| *Aquilegia coerulea* | Aquca_030_00368.1 | 55.2% (533/965) | 71.2% (687/965) | 55.2%(533/965) | 71.0%(685/965) |
| *Selaginella moellendorffii* | 77447 | 31.9% (290/909) | 48.6% (442/909) | 31.5%(288/914) | 48.5%(443/914) |
| *Physcomitrella patens* | Pp1s244_27V6.2 | 39.2% (371/946) | 57.2% (541/946) | 39.3%(358/936) | 57.8%(541/936) |
| *Panicum virgatum* | Pavirv00031150m | 76.0% (753/991) | 84.2% (834/991) | 75.4%(752/991) | 84.0%(832/991) |
| *Medicago truncatula* | Medtr5g091950.1 | 41.6%(402/967) | 60.7%(587/967) | 41.7% (404/968) | 60.4% (585/968) |
| *Glycine max* | Glyma08g25590.2 | 55.1% (532/966) | 70.1% (677/966) | 55.3% (534/966) | 70.2% (678/966) |
| *Phaseolus vulgaris* | Phvul.011G169300.1 | 57.0%(544/955) | 73.4%(701/955) | 57.2%(546/955) | 73.5%(702/955) |
