# Supplementary material for: Two members of TaRLK family confer powdery mildew resistance in common wheat
Source: BMC Plant Biol. 2016 Jan 25;16:27. doi: 10.1186/s12870-016-0713-8 (PMC4727334; doi:10.1186/s12870-016-0713-8)
Supplement: Additional file 5: Figure S2. — The schematic diagram of different types of TaRLKs. The TaRLK2 was presumed to originate from a recombination between TaRLK1 and TiRLK at the region between 396-459 bp. TaRLK Prins was also presumed to be originated from a recombination between TaRLK1 and TiRLK, but at a different region (between 1054-1125 bp). (DOC 121 kb) [file 12870_2016_713_MOESM5_ESM.doc]

**Additional file 5: Figure S2.**

**
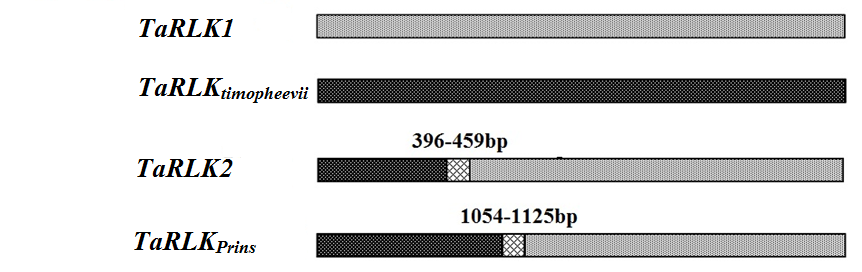
**

**Additional file 2: Figure S2.** The schematic diagram of different types of *TaRLKs*. The *TaRLK2* was presumed to originate from a recombination between *TaRLK1* and *TiRLK* at the region between 396-459 bp. *TaRLK_Prins_* was also presumed to be originated from a recombination between *TaRLK1* and *TiRLK*, but at a different region (between 1054-1125 bp)
