# Supplementary material for: Two members of TaRLK family confer powdery mildew resistance in common wheat
Source: BMC Plant Biol. 2016 Jan 25;16:27. doi: 10.1186/s12870-016-0713-8 (PMC4727334; doi:10.1186/s12870-016-0713-8)
Supplement: Additional file 6: Figure S3. — Powdery mildew resistance to Bgt isolates mixture of Prins, IGVI-465 and Yangmai158 at the first leaf stage. (DOC 761 kb) [file 12870_2016_713_MOESM6_ESM.doc]

**Additional file 6: Figure S3.**

**
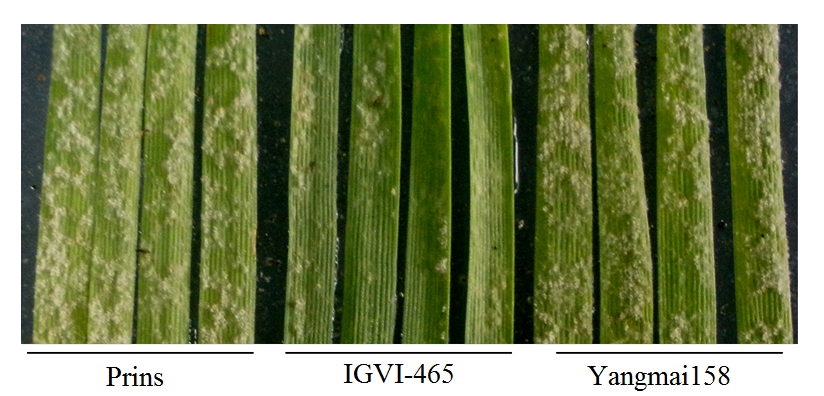
**

**Additional file 3: Figure S3.** Powdery mildew resistance to *Bgt* isolates mixture of Prins, IGVI-465 and Yangmai158 at the first leaf stage
