# Supplementary material for: Two members of TaRLK family confer powdery mildew resistance in common wheat
Source: BMC Plant Biol. 2016 Jan 25;16:27. doi: 10.1186/s12870-016-0713-8 (PMC4727334; doi:10.1186/s12870-016-0713-8)

**Additional file 7: Figure S4.** The detection of SOD, POD and CAT activities in transgenic plants at the second (left) and fourth (right) leaf growing stages after *Bgt* treatments

* indicate significant difference within each genotype at p < 0.05 level, using non-inoculated samples as control. The data were from three replicated independent experiments.


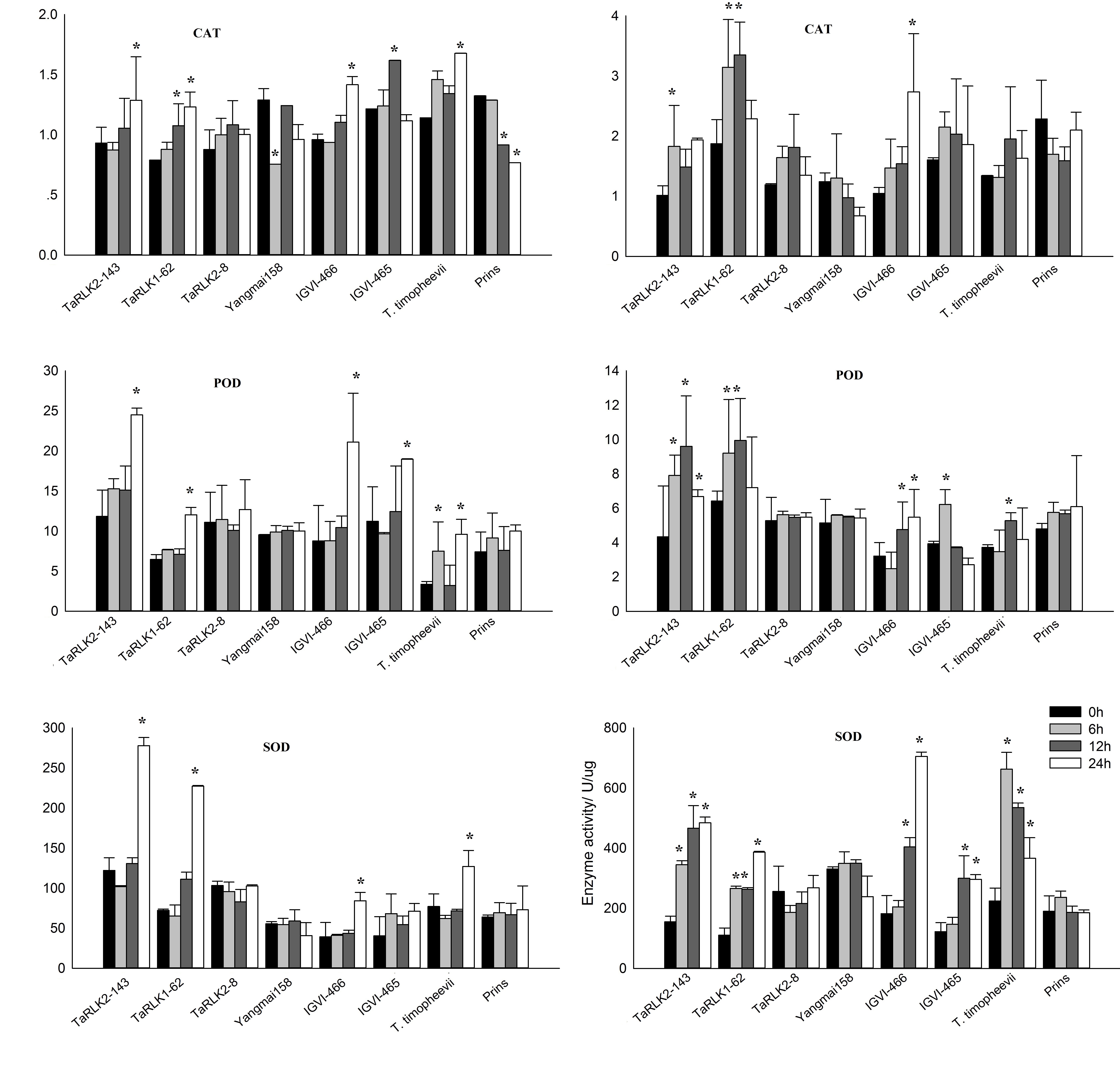

Supplement: Additional file 7: Figure S4. — The detection of SOD, POD and CAT activities in transgenic plants at the second (left) and fourth (right) leaf growing stages after Bgt treatments. * indicates significant difference within each genotypes at 0.05 levels, using non-inoculated samples as control. The data were from three replicated independent experiments. (DOC 2439 kb) [file 12870_2016_713_MOESM7_ESM.doc]
