# Supplementary material for: Two members of TaRLK family confer powdery mildew resistance in common wheat
Source: BMC Plant Biol. 2016 Jan 25;16:27. doi: 10.1186/s12870-016-0713-8 (PMC4727334; doi:10.1186/s12870-016-0713-8)
Supplement: Additional file 8: Figure S5. — Expression of four defense-related marker genes in the wild-type Yangmai 158 and TaRLK1/TaRLK2 transgenic plants without the inoculation of Bgt (* p < 0.05, ** p < 0.01). (DOC 126 kb) [file 12870_2016_713_MOESM8_ESM.doc]

**Additional file 8: Figure S5.**


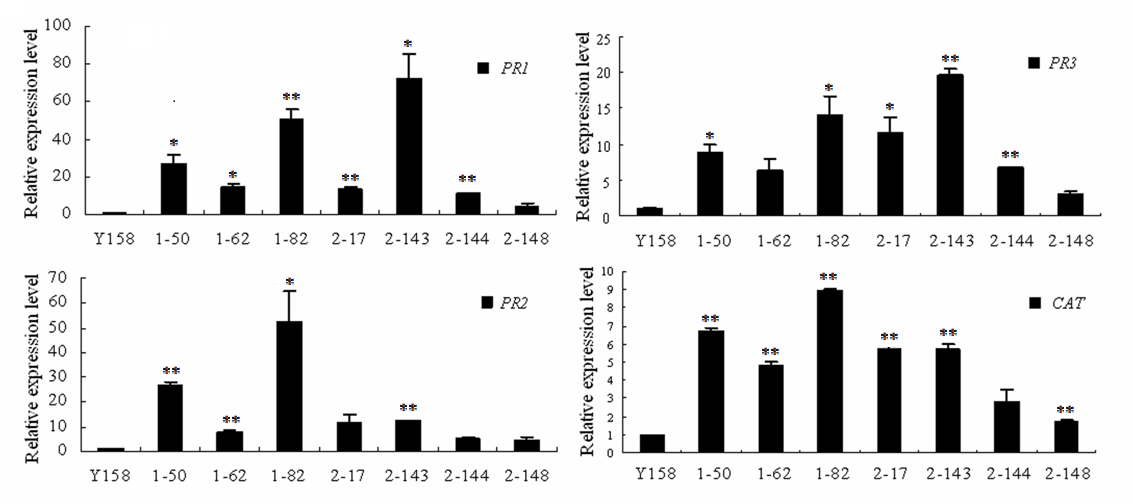


**Additional file 5: Figure S5.** Expression of four defense-related marker genes in the wild-type Yangmai 158 and *TaRLK1*/*TaRLK2* transgenic plants without the inoculation of *Bgt* (* p < 0.05, ** p < 0.01)
